# Association of 24-h urinary sodium excretion with microalbuminuria in a Chinese population

**DOI:** 10.1038/s41598-023-27874-z

**Published:** 2023-01-19

**Authors:** Chunxiao Xu, Xiaofu Du, Le Fang, Jieming Zhong, Feng Lu

**Affiliations:** Department of Chronic Non-Communicable Diseases Control and Prevention, Zhejiang Provincial Center for Disease Control and Prevention, 3399 Binsheng Road, Hangzhou, People’s Republic of China

**Keywords:** Diseases, Risk factors

## Abstract

To assess the relationship of sodium, potassium and the ratio of sodium to potassium (Na/K) with albuminuria, a cross-sectional study was carried out in China in 2017. Sodium, potassium and albumin excretions were examined in a 24-h (h) urine sample collected from 1486 participants. Microalbuminuria was defined as 24-h urinary albumin excretion between 30 and 300 mg/24 h. The participants had an average age of 46.2 ± 14.1 years old, and 48.9% were men. The proportion of patients with microalbuminuria was 9.0%. As illustrated by the adjusted generalized linear mixed model, sodium concentration increased significantly with the increase in 24-h urinary albumin (*β* = 1.16, 95% confidence interval (CI) 0.38–1.93; *P* = 0.003). Multivariable-adjusted logistic regression analyses demonstrated that the odds ratio (OR) of microalbuminuria increased with the quartiles of sodium [OR = 2.20, 95% CI 1.26–3.84 (the maximum quartile vs. the minimum quartile), *P*_for trend_ = 0.006]. Potassium and the Na/K ratio did not have any association with outcome indicators. A high amount of sodium intake was potentially correlated with early renal function impairment.

## Introduction

Increased sodium intake is a strong risk factor for hypertension and cardiovascular diseases^1^. Preclinical and clinical studies have provided evidence that high dietary salt intake may partly result in target organ damage, including albuminuria, independent of blood pressure^2,3^. Instead of the food frequency or questionnaire method, the 24-h (h) urinary sodium method is used for precise and in-depth studies, as it is not limited by the high variability or recall bias of dietary intake patterns.

Urinary albumin is a crucial indicator of renal impairment in an early stage, which is a strong risk factor for cardiovascular complications in the general population^4^. The 24-h urinary albumin excretion rate is regarded as the gold standard for measuring albuminuria. However, most of the literature has adopted the albumin/creatine ratio as an alternative due to convenience and cost^5,6^.

The relationships of sodium and potassium with albuminuria have not been fully elucidated, and findings are not consistent in the literature. Several studies^7,8^ have shown that sodium intake is positively correlated with urinary albumin. In contrast, there was no vital relationship between sodium and albuminuria in several cross-sectional studies^9,10^.

This study aimed to investigate the relationships of 24-h urinary sodium and potassium excretion and the sodium-to-potassium ratio (Na/K ratio) with microalbuminuria in a Chinese population from Zhejiang Province.

## Materials and methods

### Study design

This study used baseline survey data from a cross-sectional study in Zhejiang Province in 2017^11^. Eighteen- to 69-year-old subjects were randomly selected by a stratified multistage randomized sampling method in Zhejiang Province. Finally, 3 urban areas and 2 rural areas in eastern, northeastern, central, middle western, and southern Zhejiang Province were selected for the study. The Ethics Review Committee of Zhejiang Provincial Center for Disease Control and Prevention (CDC) favored this study. All participants signed informed consent forms. All methods, including biological specimen examination, anthropometric measurement and questionnaire surveys, were conducted according to relevant guidelines and regulations.

### Main outcomes and measures

Questionnaire data included demographic characteristics, history of chronic diseases including hypertension, diabetes and cardiovascular disease (CVD), and lifestyles including smoking, drinking, and physical activities. Self-reported CVD history included stroke and coronary heart disease. Diabetes and chronic kidney disease (CKD) were approved by a health care provider's diagnosis or based on medication use.

Current smoking is defined as smoking > 1 cigarette a day for 6 months. Alcohol drinking status is defined as drinking at least 1 time a week in the past year. Self-reported physical activity is defined as ≥ 150 min (min) of moderate-intensity physical activity per week or a combination of moderate- and high-intensity physical activity per week or ≥ 75 min of high-intensity physical activity per week. The definition of hypertension is systolic blood pressure (SBP) ≥ 140 mm Hg and/or diastolic blood pressure (DBP) ≥ 90 mm Hg and/or self-reported use of antihypertensive medication within two weeks^12^. The measurements of blood pressure, weight, and waist circumference were described elsewhere^11^.

Sodium intake was evaluated by 24-h urinary sodium excretion. The ion selective electrode method (C16000, Abbott Corp., America) was used for the analyses of sodium and potassium. Urinary creatinine was measured by the picric acid method, and an immuno nephelometric method was used to measure 24-h urinary albumin excretion (C501, Roche Cob as Corp., Switzerland). Urinary excretion was the cross-product of the analyte concentration multiplied by the volume of urine in 24 h.

Subjects who had unqualified urine collections were excluded from the analyses. The exclusion criteria were as follows: 24-h urinary volume < 500 mL; acquisition time less than 22 h; urinary creatinine excretion ± 2 standard deviations from the exclusion of sex specificity^13^; and a reported history of chronic kidney disease. A total of 1486 qualified specimens were included.

Microalbuminuria was diagnosed based on the amount of 24-h urinary albumin excretion in the range of 30–300 mg/24-h^14^.

### Statistical analysis

Continuous data were expressed as the mean (M) ± standard deviation (SD), while categorical data were presented as frequency (n) and percentage (%). A *t* test or chi-squared test was adopted to compare demographic, socioeconomic and clinical variables across subjects with and without microalbuminuria.

Linear mixed models were employed for the associations of urinary albumin level with the sodium, potassium (each 1000 mg), and Na/K ratio (each 1-unit molar ratio). A multivariable logistic regression model was employed to estimate the odds ratio (OR) and 95% confidence interval (CI) for the associations of microalbuminuria with the quartiles of sodium, potassium or Na/K ratio under the circumstance of the lowest quartile as a reference group, and linear trends were tested by calculating the index of sodium, potassium and Na/K ratio as a continuous variable. Two covariate-adjusted models were used in both linear and logistic regression analyses.

Model 1 was adjusted for sex and age, and model 2 was further adjusted for BMI, SBP, alcohol consumption, smoking, physical activities, diabetes, and antihypertensive medication use.

Sensitivity analyses were performed to exclude subjects diagnosed with CVD or taking any drugs, including anti-hypertension, diuretics, vasodilators and sedative drugs, on the interview day, as well as those who met both of the above conditions. Two-side *P* values < 0.05 were considered significant. Analyses were performed using the SAS statistical package (version 9.2; SAS Institute, Inc., Cary, North Carolina, USA).

## Results

The baseline characteristics of 1486 participants with or without microalbuminuria are given in Table 1. The age of the participants was 46.2 ± 14.1 years old, and 48.9% were men. The proportion of microalbuminuria was 9.0%. Different from the participants without microalbuminuria, those with microalbuminuria had a higher possibility of being drinkers, had a higher waist circumference and higher serum triglyceride (TG) level, had higher sodium intake and were more likely to develop hypertension, diabetes, obesity and dyslipidemia.

**Table 1:** Characteristics of 1486 participants by the status of microalbuminuria in a cross-sectional study in China in 2017.

Characteristic	All (n = 1486)	Microalbuminuria		*P*
		No (n = 1352)	Yes (n = 134)	
Age (years)	46.2 ± 14.1	46.1 ± 14.1	47.9 ± 13.4	0.158
Male, n (%)	726 (48.9)	649 (48.0)	77 (57.5)	0.037
Current smoking, n (%)	336 (22.6)	296 (21.9)	40 (29.9)	0.061
Drinking, n (%)	486 (32.7)	431 (31.9)	55 (41.0)	0.031
Education years, n (%)				0.973
< 9 years	479 (32.2)	437 (32.3)	42 (31.3)	
9–12 years	688 (46.3)	625 (46.2)	63 (47.0)	
> 12 years	319 (21.5)	290 (21.4)	29 (21.6)	
Physical activity, n (%)	607 (40.8)	546 (40.4)	61 (45.5)	0.248
BMI, n (%)				0.028
< 24	786 (52.9)	730 (54.0)	56 (41.8)	
24–28	532 (35.8)	477 (35.3)	55 (41.0)	
≥ 28	168 (11.3)	145 (10.7)	23 (17.2)	
WC (cm)	81.4 ± 9.5	81.0 ± 9.3	84.5 ± 10.7	< 0.001
SBP (mmHg)	129.6 ± 19.4	128.8 ± 19.2	137.5 ± 20.3	< 0.001
DBP (mmHg)	79.9 ± 10.9	79.4 ± 10.7	84.6 ± 12.1	< 0.001
FPG (mmol)	5.2 ± 1.3	5.2 ± 1.3	5.6 ± 1.8	< 0.001
Sodium (mg/24 h)	3849.8 ± 1661.7	3809.5 ± 1654.0	4263.2 ± 1690.8	0.003
Potassium (mg/24 h)	1491.2 ± 711.8	1481.8 ± 711.2	1587.0 ± 713.5	0.105
NA/K ratio	4.9 ± 2.4	4.9 ± 2.4	5.1 ± 2.5	0.296
24-h creatinine, mg/24 h	10.1 ± 4.8	10.0 ± 4.7	11.1 ± 5.5	0.010
24-h urine volume, ml/24 h	1446.8 ± 446.5	1433.9 ± 441.9	1579.0 ± 472.7	< 0.001
Serum LDL-C (mmol/L)	2.7 ± 0.7	2.7 ± 0.7	2.8 ± 0.8	0.295
Serum TG (mmol/L)	1.5 ± 1.3	1.5 ± 1.3	1.8 ± 1.8	0.005
Dyslipidemia, n (%)	508 (34.2)	448 (33.1)	60 (44.8)	0.007
Hypertension, n (%)	525 (35.5)	451 (33.4)	74 (55.2)	< 0.001
Anti-hypertensive drugs, n (%)	198 (13.4)	170 (12.6)	28 (21.2)	0.005
Diabetes mellitus, n (%)	132 (8.9)	108 (8.0)	24 (17.9)	< 0.001

In the adjusted linear regression models, 24-h sodium excretion was significantly related to 24-h urinary albumin excretion (1.16 mg/24 h; 95% CI 0.38–1.93) for each 1-g increase in sodium excretion (Table 2). There was no significant association of potassium or the Na/K ratio with 24-h urinary albumin excretion in any model.

**Table 2:** Associations of 24-h urinary sodium, potassium excretion (g/24 h), and their ratio with a 24-h urinary albumin excretion (mg/24 h) using the mixed linear effect models.

Factor in two models	*β*-coefficient	95% CI	*P*
Sodium excretion, g/24 h			
Model 1	1.35	0.57–2.13	0.001
Model 2	1.16	0.38–1.93	0.003
Potassium excretion, g/24 h			
Model 1	1.98	0.19–3.78	0.030
Model 2	1.76	− 0.03–3.55	0.053
Sodium-to-potassium ratio			
Model 1	0.27	− 0.27–0.81	0.328
Model 2	0.21	− 0.34–0.75	0.458

Model 1 adjusted for age and sex.

Model 2 adjusted for age, sex, smoking, drinking, physical activity, BMI, SBP, fasting blood glucose, dyslipidemia and use of antihypertensive drugs.

Table 3 presents the adjusted OR and 95% CI of microalbuminuria in the multivariable adjusted logistic model with the lowest quartile as a reference for sodium, potassium and the Na/K ratio. After completely adjusting for the confounding factors in model 2, the ORs of the fourth quartile of sodium were still significantly associated with microalbuminuria (OR = 2.20, 95% CI: 1.26–3.84). The ORs of potassium and the Na/K ratio quartiles remained insignificant in all models.

**Table 3:** Associations of each quartile (Q) of 24-h urinary sodium, potassium excretion, and their ratio with microalbuminuria in a cross-sectional study in China in 2017.

Factors	Model 1		Model 2	
	OR	95% CI	OR	95% CI
Sodium excretion, (mg/24 h)				
Q1	1.00		1.00	
Q2	1.58	0.89–2.81	1.56	0.87–2.80
Q3	1.68	0.95–2.96	1.67	0.94–2.98
Q4	2.37	1.37–4.09	2.20	1.26–3.84
*P* for trend		0.002		0.006
Potassium excretion, mg/24 h				
Q1	1.00		1.00	
Q2	1.24	0.73–2.12	1.39	0.80–2.40
Q3	1.47	0.87–2.48	1.52	0.89–2.61
Q4	1.50	0.89–2.53	1.66	0.97–2.84
*P* for trend		0.117		0.100
Sodium-to-potassium ratio				
Q1	1.00		1.00	
Q2	0.86	0.50–1.47	0.74	0.43–1.28
Q3	1.47	0.90–2.39	1.29	0.78–2.13
Q4	0.86	0.50–1.48	0.71	0.41–1.24
*P* for trend		0.911		0.565

Q indicates quartile, OR indicates odds ratio, CI indicates confidence interval;

Model 1 adjusted for age and sex;

Model 2 adjusted for age, sex, smoking, drinking, physical activity, BMI, hypertension, diabetes, dyslipidemia and antihypertensive medication use.

**Figure 1:**
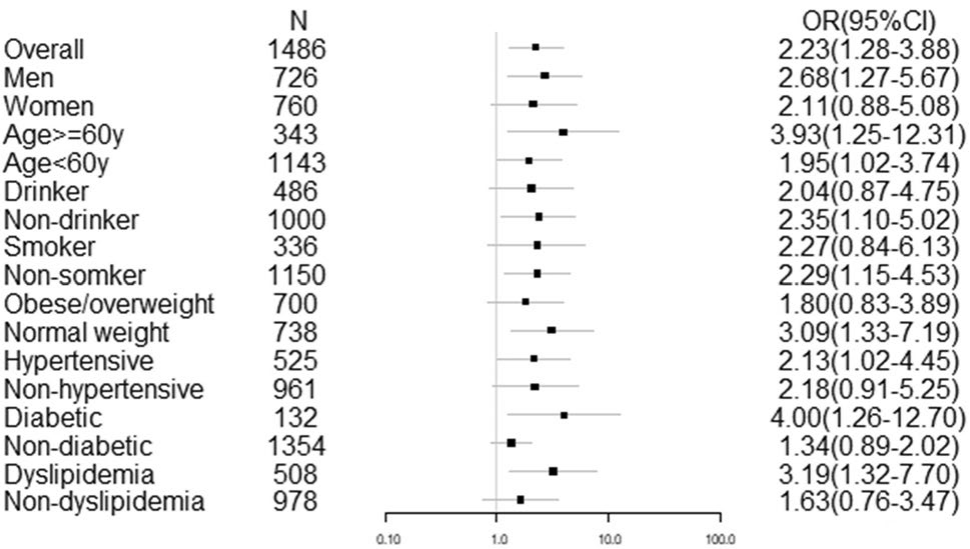
The relationship between the maximum quartile of urinary sodium excretion and microalbuminuria: odds ratio (OR) with 95% confidence interval (CI) in the whole study and in selected subgroups. Analyses were controlled for age, sex, smoking, drinking, physical activity, BMI, hypertension, diabetes, dyslipidemia, and use of antihypertensive drugs.

Table 4 and Fig. 1 show that the results of sodium and microalbuminuria are consistent and significant in subgroup analyses for subjects who are male, age ≥ 60 years and < 60 years, nondrinkers, nonsmokers, normal weight, hypertensive, diabetic, dyslipidemia, and use of antihypertensive drugs. No interaction between variables was observed.

**Table 4:** Odds ratio (OR) and 95% confidence interval (CI) of the highest quartile of 24-h urinary sodium excretion with microalbuminuria in subgroup analyses.

Factors	Number	OR	95% CI	*P*_for interaction_
Overall	1486	2.20	1.26–3.84	
Gender				0.981
Men	726	2.61	1.23–5.56	
Women	760	2.23	0.92–5.38	
Age				0.311
≥ 60 y	343	3.91	1.24–12.29	
< 60 y	1143	1.92	1.00–3.70	
Drinker				0.305
Yes	486	2.04	0.87–4.79	
No	1000	2.33	1.08–5.01	
Smoker				0.568
Yes	336	2.02	0.73–5.58	
No	1150	2.37	1.19–4.72	
BMI				0.651
Obese/overweight	700	1.78	0.82–3.87	
Normal weight	738	2.99	1.28–6.97	
Hypertension				0.782
Yes	525	2.19	1.05–4.58	
No	961	2.13	0.89–5.12	
Use of antihypertensive drugs				0.406
Yes	213	3.08	1.01–9.41	
No	1273	1.85	0.95–3.60	
Diabetes				0.143
Yes	132	3.89	1.23–12.31	
No	1354	1.31	0.87–1.98	
Dyslipidemia				0.377
Yes	508	3.11	1.28–7.53	
No	978	1.61	0.75–3.43

The sensitivity analyses showed no change in ORs between sodium and microalbuminuria after exclusion of patients with CVD or using any drugs, including anti-hypertensive drugs, diuretics, vasodilators and sedative drugs, on the interview day, as well as those who met the above conditions (Table 5).

**Table 5:** Sensitivity analyses: Adjusted odds ratios (OR) and 95% confidence intervals (CI) for microalbuminuria according to the quartiles of the 24-h urinary sodium excretion.

Sodium excretion (mg/24 h)	Model 1		Model 2	
	OR	95% CI	OR	95% CI
n = 1452 after excluding group A				
Q1	1.00		1.00	
Q2	1.48	0.82–2.68	1.46	0.80–2.66
Q3	1.78	1.01–3.17	1.76	0.98–3.16
Q4	2.29	1.31–4.00	2.13	1.20–3.76
*P* for trend	0.003		0.008	
n = 1359 after excluding group B				
Q1	1.00		1.00	
Q2	1.78	0.94–3.37	1.80	0.94–3.44
Q3	1.93	1.03–3.61	1.92	1.01–3.63
Q4	2.44	1.32–4.50	2.38	1.27–4.44
*P* for trend	0.006		0.010	
n = 1332 after excluding groups A and B				
Q1	1.00		1.00	
Q2	1.55	0.81–2.98	1.56	0.81–3.02
Q3	1.92	1.03–3.60	1.91	1.01–3.62
Q4	2.19	1.18–4.07	2.12	1.13–3.99
*P* for trend	0.012		0.020	

Group A with cardiovascular disease (stroke, coronary heart disease);

Group B using anti-hypertensive drugs, diuretics, vasodilators and sedative drugs on the interview day.

## Discussion

This study reports the cross-sectional relationship between 24-h urinary sodium and microalbuminuria in a population-based study in China, which is independent of many other variables, especially blood pressure and antihypertensive drugs.

In the Prevention of Renal and Vascular End Stage Disease (PREVEND) study^7^, the researchers found that after adjusting for confounders, when the sodium intake increased by 1 g, the urinary albumin excretion increased by 4.6 mg/d; In the current study, every 1-g increase in sodium led to an increase in urinary albumin excretion by 1.16 mg/d. This difference may depend on the characteristics of the study participants. Studies by du Cailar et al.^15^, Sun et al.^3^ and Xu et al.^16^ revealed that 24-h urinary sodium is a strong and independent determinant of urinary albumin excretion. In several studies, a significant correlation between sodium intake and proteinuria was found regardless of blood pressure, which demonstrated that blood pressure was not the only reason for the harmful sodium effect.

On the other hand, our study showed that potassium intake and the Na/K ratio had no relationship with microalbuminuria. Previous studies have drawn controversial conclusions on the associations between potassium intake, the Na/K ratio and albuminuria. In the Reasons for Geographic and Racial Differences in Stroke (REGARDS) study based on food frequency questionnaires, no association of albuminuria with potassium intake was found^8^. A recent study found that higher 24-h urinary potassium excretion was associated with a lower risk for renal dysfunction^17^, and another study showed that higher dietary potassium intake was associated with kidney disease progression in CKD patients^18^. The Na/K ratio may be an important indicator of CKD and estimated glomerular filtration rate (eGFR) decline in addition to sodium or potassium alone^19^. However, relevant findings on the impact of the Na/K ratio on kidney function are limited. A cohort study showed that estimated 24-h urinary sodium excretion and the Na/K ratio from spot urine were predictors of kidney function decline measured by eGFR^20^. Another cohort study found that a high self-reported dietary Na/K ratio was associated with an increased risk of incident CKD^19^. A cross-sectional study in China concluded that a high urinary Na/K ratio was associated with albuminuria, a sign of early renal impairment^3^. In contrast, the Shangdong-Ministry of Health Action on Salt and Hypertension (SMASH) study conducted in Chinese individuals found no association between urinary potassium or the Na/K ratio and albuminuria in a general population^21^. Furthermore, a longitudinal Japanese study did not recommend the Na/K ratio from spot urine as a good marker in assessing renal function decline^22^. These inconsistent conclusions may be due to different characteristics of the study population, different measures of sodium and potassium (i.e., spot urine collections, single 24-h urine collections, and food frequency questionnaires) and different indicators of kidney function assessed.

Furthermore, low potassium intake in China might be part of the reason for this phenomenon^23^. Nearly all of the participants in the study had potassium intake below the World Health Organization (WHO)’s recommended minimum of 3.5 g/d^24^. Individuals who had a higher mean urinary sodium excretion may not have lower mean urinary potassium excretion. Therefore, it was unexpected that no significant association was observed between urinary potassium, the Na/K ratio and albuminuria in this Chinese population-based study. Further exploration of this issue in our future cohort studies with more repeated measurements should be conducted.

The biological mechanism of the effect of sodium and potassium on kidney function remains unclear. The positive correlation between urinary sodium excretion and urinary albumin excretion may be due to the adverse effect of sodium on the arterial vessel wall^25^. Increased urinary albumin loss is considered to be a result of endothelial injury. Endothelial injury may lead to increased susceptibility to cardiovascular and renal diseases in subjects with microalbuminuria. High potassium intake was shown to reduce vascular resistance, decrease blood pressure and increase eGFR by directly improving kidney function and therefore may play a protective role in the incidence of albuminuria^19^.

Drugs such as angiotensin receptor blockers, diuretics, and angiotensin-converting enzyme inhibitors can have a great influence on 24-h urinary sodium levels. The use of these drugs has a greater possibility of occurring in people with the highest risk of CVD, so the potential for measurement error is greater^26^. Therefore, people with CVD and those who used any antihypertensive drugs, diuretics, vasodilators or sedative drugs were excluded from the sensitivity analyses to assess study robustness.

The strengths of the study include a large randomized sample of the Chinese population, completion of follow-up, a relatively robust method to evaluate population sodium intake and 24-h urinary albumin excretion through 24-h urine collection, an adequate sample size, and a rigorous approach to control measurement errors.

Our study has some limitations. The results were based on only one-time 24-h urine collection to minimize the burden for participants and the cost for our study. The potential individual differences in sodium intake may be overestimated, and therefore, the advantage of sodium and potassium intake related to albuminuria might be underestimated. Finally, this is a cross-sectional study, although the results were adjusted for major confounders.

In summary, our study showed independent and positive associations of high sodium excretion with microalbuminuria in the general population. With several preventive measures adopted, health-oriented policies for the purpose of reducing sodium intake in the population can relieve not only the burden of albuminuria but also other related disorders.

## Data Availability

The data that support the findings of our study are not publicly available due to the issue of copyright but are available from the corresponding author upon reasonable request.

## References

[1] Morrison AC, Ness RB (2011). Sodium intake and cardiovascular disease. Annu. Rev. Public Health..

[2] Jardine MJ (2019). Dietary sodium reduction reduces albuminuria: A cluster randomized trial. J. Ren. Nutr..

[3] Sun Y (2021). Association of sodium, potassium and sodium-to-potassium ratio with urine albumin excretion among the general Chinese population. Nutrients.

[4] Pedrinelli R, Dell'Omo G, Di Bello V, Pontremoli R, Mariani M (2002). Microalbuminuria, an integrated marker of cardiovascular risk in essential hypertension. J. Hum. Hypertens..

[5] Cirillo M, Cavallo P, Zulli E, Villa R, Veneziano R, Costanzo S (2021). Sodium intake and proteinuria/albuminuria in the population-observational, cross-sectional study. Nutrients.

[6] Elfassy T, Zhang L, Raij L, Bibbins-Domingo K, Lewis CE, Allen NB, Liu KJ, Peralta CA, Odden MC, Zeki AA (2020). Results of the CARDIA study suggest that higher dietary potassium may be kidney protective. Kidney Int..

[7] Forman JP, Scheven L, de Jong PE, Bakker SJ, Curhan GC, Gansevoort RT (2012). Association between sodium intake and change in uric acid, urine albumin excretion, and the risk of developing hypertension. Circulation.

[8] Aaron KJ, Campbell RC, Judd SE, Sanders PW, Muntner P (2011). Association of dietary sodium and potassium intakes with albuminuria in normal-weight, overweight, and obese participants in the reasons for geographic and racial differences in stroke (REGARDS) study. Am. J. Clin. Nutr..

[9] Kieneker LM, Bakker SJ, de Boer RA, Navis GJ, Gansevoort RT, Joosten MM (2016). Low potassium excretion but not high sodium excretion is associated with increased risk of developing chronic kidney disease. Kidney Int..

[10] Sharma S, McFann K, Chonchol M, de Boer IH, Kendrick J (2013). Association between dietary sodium and potassium intake with chronic kidney disease in US adults: A cross-sectional study. Am. J. Nephrol..

[11] Du X (2021). Use of salt-restriction spoons and its associations with urinary sodium and potassium in the Zhejiang Province of China: Results of a population-based survey. Nutrients.

[12] Mancia G (2013). 2013 ESH/ESC Guidelines for the management of arterial hypertension: the Task Force for the management of arterial hypertension of the European Society of Hypertension (ESH) and of the European Society of Cardiology (ESC). J. Hypertens..

[13] Mann SJ, Gerber LM (2019). Addressing the problem of inaccuracy of measured 24-hour urine collections due to incomplete collection. J. Clin. Hypertens (Greenwich)..

[14] Crews DC, Boulware LE, Gansevoort RT, Jaar BG (2011). Albuminuria: Is it time to screen the general population?. Adv. Chronic Kidney Dis..

[15] du Cailar G, Ribstein J, Mimran A (2002). Dietary sodium and target organ damage in essential hypertension. Am. J. Hypertens..

[16] Xu JW, Wu J, Chen XR, Yan LX, Cai XN, Ma JX (2019). Association between 24 h urinary sodium excretion and microalbuminuria among Chinese people aged from 18 to 69 years old. Zhonghua Yu Fang Yi Xue Za Zhi.

[17] Araki S, Haneda M, Koya D, Kondo K, Tanaka S, Arima H (2015). Urinary potassium excretion and renal and cardiovascular complications in patients with type 2 diabetes and normal renal function. Clin. J. Am. Soc. Nephrol..

[18] Lin J, Hu FB, Curhan GC (2010). Associations of diet with albuminuria and kidney function decline. Clin. J. Am. Soc. Nephrol..

[19] Swift SL, Drexler Y, Sotres-Alvarez D, Raij L, Llabre MM, Schneiderman N (2022). Associations of sodium and potassium intake with chronic kidney disease in a prospective cohort study: Findings from the Hispanic Community Health Study/Study of Latinos, 2008–2017. BMC Nephrol..

[20] Deriaz D, Guessous I, Vollenweider P, Devuyst O, Burnier M, Bochud M, Ponte B (2019). Estimated 24-h urinary sodium and sodium-to-potassium ratio are predictors of kidney function decline in a population-based study. J. Hypertens..

[21] Yan L, Guo X, Wang H, Zhang J, Tang J, Lu Z (2016). Population-based association between urinary excretion of sodium, potassium and its ratio with albuminuria in Chinese. Asia Pac. J. Clin. Nutr..

[22] Tabara Y, Takahashi Y, Setoh K, Kawaguchi T, Kosugi S, Nakayama T, Matsuda F, Nagahama Study Group (2017). Prognostic significance of spot urine Na/K for longitudinal changes in blood pressure and renal function: The Nagahama study. Am. J. Hypertens..

[23] Tan M, He FJ, Wang C, MacGregor GA (2019). Twenty-four-hour urinary sodium and potassium excretion in China: A systematic review and meta-analysis. J. Am. Heart Assoc..

[24] Guideline: Potassium Intake for Adults and Children. Geneva: World Health Organization (2012) 23617019

[25] Patik JC, Lennon SL, Farquhar WB, Edwards DG (2021). Mechanisms of dietary sodium-induced impairments in endothelial function and potential countermeasures. Nutrients.

[26] Aaron KJ, Sanders PW (2013). Role of dietary salt and potassium intake in cardiovascular health and disease: A review of the evidence. Mayo Clin. Proc..

